# Supplementary material for: Monoclonal Antibody Therapy for COVID-19: A Retrospective Observational Study at a Regional Hospital
Source: Infect Dis Rep. 2023 Feb 20;15(1):125–31. doi: 10.3390/idr15010013 (PMC9956015; doi:10.3390/idr15010013)
Supplement: Supplementary file 1 [file idr-15-00013-s001.zip › Fig S1 patient recruitment.pdf]

Figure S1: Recruited patients and treatments in this study.

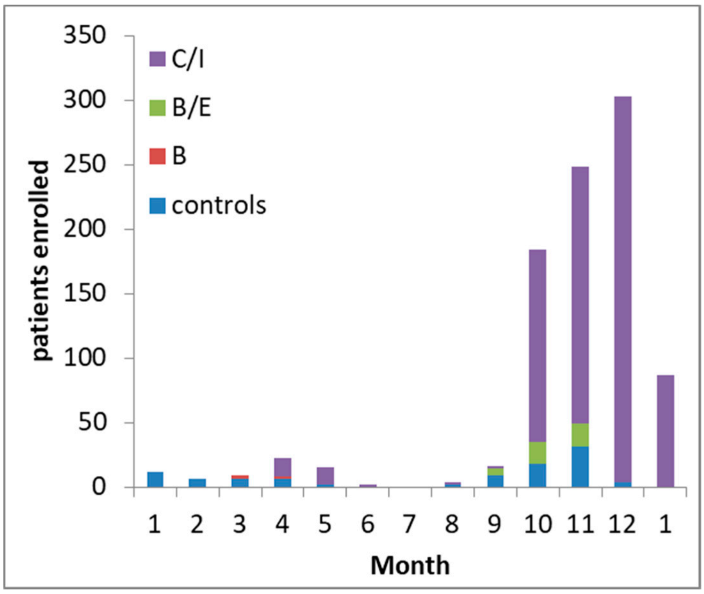

Time period	Number patients	mAb
January 22 – May 22	32	none
March 22 – April 22	5	B
April 22 – January 23	618	C/I
September 22 – November 22	40	B/E
August 22 – December 22	64	none

B: Bamlanivimab

C/I: Casirivimab/Imdevimab

B/E: Bamlanivimab/ Etesevimab
