# Supplementary material for: Monoclonal Antibody Therapy for COVID-19: A Retrospective Observational Study at a Regional Hospital
Source: Infect Dis Rep. 2023 Feb 20;15(1):125–31. doi: 10.3390/idr15010013 (PMC9956015; doi:10.3390/idr15010013)
Supplement: Supplementary file 1 [file idr-15-00013-s001.zip › Table S1.pdf]

Table S1: Statistical analysis of clinical parameters for all patients included. Missing data were excluded, causing different numbers for each parameter. Average ± standard deviation is shown. For metric parameters significance was determined using Student´s T-test, Cross tables were analysed using Fisher´s exact test.$p < 0.1$: +; $p < 0.05$: *; $p < 0.01$: **

**Parameter**	**All patients**		
	All	untreated	treated
Number	752	98	654
Age	64.7 ± 16.9	65.6 ± 21.2	64.6 ± 16.2
**Symptoms**			
Coughing (n/y)<br>% yes	292/410<br>58.4 %	54/36<br>40.0 %	238/374 **<br>61.1 %
Dyspnea (n/y)<br>% yes	529/170<br>24.3 %	47/43<br>47.8 %	482/172 **<br>26.3 %
Fatigue (n/y)	510/187<br>26.8%	69/21<br>23.3 %	441/166<br>27.3 %
Pain (n/y)<br>% yes	451/248<br>35.5 %	70/20<br>22.2 %	381/228 **<br>37.4 %
Inappetence (n/y)<br>% yes	600/99<br>14.2 %	76/14<br>15.6 %	524/85<br>14.0 %
Loss of taste and smell (n/y)<br>% yes	637/61<br>8.7 %	86/4<br>4.4 %	551/57<br>9.4 %
Diarrhoea / vomitting (n/y)<br>% yes	612/88<br>12.6 %	81/9<br>10.0 %	531/79<br>13.0 %
Fever (n/y)<br>% yes	478/221<br>31.6 %	54/36<br>40.0 %	424/185 +<br>30.4 %
Temperature	37.3 ± 1.0	37.3 ± 1.0	37.3 ± 1.0
Neurological Symptoms (n/y) % yes	656/42<br>6.0 %	84/6<br>6.7 %	572/36<br>5.9 %
Syncope (n/y)<br>% yes	669/30<br>4.3 %	80/10<br>11.1 %	589/20 **<br>3.3 %
**Preexisting illness**			
Hypertension (n/y)<br>% yes	314/386<br>55.1 %	44/49<br>52.7 %	270/337<br>55.5 %
Blood pressure systolic	128.3 ± 19.0	125 ± 2.8	128.7 ± 0.8
Blood pressure diastolic	77.9 ± 12.3	73.6 ± 11.7	78.4 ± 12.3 **
Cardiac frequency (1/min)	81.7 ± 16.2	84.3 ± 1.9	81.0 ± 1.0
Diabetes (n/y)<br>% yes	547/154<br>22.0 %	69/24<br>25.8 %	478/130<br>21.4 %
Renal insufficency (n/y)<br>% yes	586/113<br>16.2 %	73/20<br>21.5 %	513/93<br>15.3 %
COPD/Asthma (n/y)<br>% yes	569/130<br>18.6 %	72/21<br>22.6 %	497/109<br>18.0 %
Active malignoma (n/y)<br>% yes	640/61<br>8.7 %	86/7<br>7.5 %	554/54<br>8.9 %
Inactive malignoma (n/y)<br>% yes	664/37<br>5.3 %	86/6<br>6.5 %	578/28<br>4.6 %
Immunosuppression (n/y)<br>% yes	661/39<br>5.6 %	90/3<br>3.2 %	571/36<br>5.9 %
Obesity (n/y)	560/137	73/19	487/118

% yes	19.7 %	20.7 %	19.5 %
Heart disease (n/y)<br>% yes	522/174<br>25 %	62/31<br>33.3 %	460/143 +<br>23.7 %
Hypothyreosis (n/y)<br>% yes	608/88<br>12.6 %	76/16<br>17.4 %	532/72<br>11.9 %
**Blood gas analysis**			
pO_2	10.2 ± 2.7	9.7 ± 4.4	10.3 ± 2.3
pCO_2	4.9 ± 2.0	4.7 ± 0.9	5.0 ± 2.1
O_2-Saturation %	95.2 ± 30.8	92.5 ± 4.9	95.6 ± 33.1
**Clinical chemistry**			
Hemoglobin (mM)	8.4 ± 4.0	8.2 ± 1.2	8.4 ±1.3 +
Leukocytes (Gpt/L)	6.5 ± 8.0	7.5 ± 0.4	6.0 ± 4.5 **
Lymphocytes (Gpt/L)	1.6 ± 2.2	1.5 ± 2.2	1.6 ± 2.2
Thrombocytes (Gpt/L)	205.5 ± 78.5	204.9± 88.6	206.6 ± 76.3
CRP (mg/L)	39.5 ± 52.7	69.2± 69.3	34.4 ± 47.6 **
proBNP (mg/mL)	2149 ± 4393	2413 ± 5191	2025 ± 3981
Troponin (ng/mL)	0.045 ± 0.16	0.08 ± 0.30	0.036 ± 0.09
Blood glucose (mM)	8.1 ± 9.4	9.6 ± 0.3	7.6 ± 0.1
Creatinin(µM)	106.4 ± 106.5	111.3 ± 111.4	105.6 ± 105.8
GFR (mL/min/1.73m²)	74.2 ± 28.1	72.4 ± 34.0	74.5 ± 27.0
RT-PCR (Ct)	24.7 ± 5.2	26.7 ± 4.9	24.3 ± 5.2 **
Hospitalization (d)	5.9 ± 9.0	14.4 ± 11.1	4.5 ± 7.8 **
Death (n/y)<br>% yes	622/40<br>6.0 %	77/17<br>18.1 %	545/23 **<br>4.1 %

**Parameter**		**Female**	
	All	untreated	treated
Number	406	59	347
Age	66.3 ± 17.1	64.7 ± 21.0	66.6 ± 13.3
**Symptoms**			
Coughing (n/y)<br>% yes	153/218<br>58.7 %	31/22<br>41.5 %	122/196 **<br>61.6 %
Dyspnea (n/y)<br>% yes	275/96<br>25.9 %	25/28<br>52.8 %	250/68 **<br>21.4 %
Fatigue (n/y)	269/101<br>27.3 %	37/16<br>30.2 %	232/85<br>26.8 %
Pain (n/y)<br>% yes	234/136<br>36.8 %	38/15<br>28.3 %	196/121<br>38.2 %
Inappetence (n/y)<br>% yes	311/60<br>16.2 %	44/9<br>17.0 %	267/51<br>16.0 %
Loss of taste and smell (n/y)<br>% yes	338/32<br>8.6 %	52/1<br>1.9 %	286/31 +<br>9.8 %
Diarrhoea / vomitting (n/y)<br>% yes	313/58<br>15.6 %	46/7<br>13.2 %	267/51<br>16.0 %
Fever (n/y)<br>% yes	255/116<br>31.2 %	31/22<br>41.5 %	224/94<br>29.6 %
Temperature	37.3 ± 1.0	37.3 ± 1.0	37.4 ± 1.0
Neurological Symptoms	345/26	49/4	296/22

(n/y) % yes	7.0 %	7.5 %	6.9 %
Syncope (n/y)<br>% yes	355/16<br>4.3 %	46/7<br>13.2 %	309/9 **<br>2.8 %
**Preexisting illness**			
Hypertension (n/y)<br>% yes	172/197<br>53.4 %	27/28<br>50.9 %	145/169<br>53.8 %
Blood pressure systolic	127.5 ± 20.4	123.9 ± 24.4	128.0 ± 19.8
Blood pressure diastolic	76.8 ± 12.6	72.8 ± 12.4	77.6 ± 12.3 *
Cardiac frequency (1/min)	82.1 ± 15.7	83.7 ± 15.5	81.6 ± 16.0
Diabetes (n/y)<br>% yes	298/71<br>19.2 %	44/11<br>20.0 %	254/60<br>19.2 %
Renal insufficency (n/y)<br>% yes	308/61<br>16.5 %	45/10<br>18.2 %	263/51<br>16.2 %
COPD/Asthma (n/y)<br>% yes	298/71<br>19.2 %	44/11<br>20.0 %	154/60<br>29.4 %
Active malignoma (n/y)<br>% yes	344/25<br>6.8 %	53/2<br>3.6 %	291/23<br>7.3 %
Inactive malignoma (n/y)<br>% yes	351/15<br>4.1 %	50/5<br>9.1 %	301/10 +<br>3.2 %
Immunosuppression (n/y)<br>% yes	346/23<br>6.2 %	54/1<br>1.8 %	292/22<br>7.0 %
Obesity (n/y)<br>% yes	290/79<br>21.4 %	40/15<br>27.3 %	250/64<br>20.4 %
Heart disease (n/y)<br>% yes	285/81<br>22.1 %	39/16<br>29.0 %	246/65<br>20.9 %
Hypothyreosis (n/y)<br>% yes	301/65<br>17.8 %	42/13<br>23.6 %	259/52<br>16.7 %
**Blood gas analysis**			
pO_2	10.2 ± 2.3	9.1 ± 2.2	10.4 ± 2.3 **
pCO_2	4.8 ± 0.7	4.8 ± 0.9	4.9 ± 0.7
O_2-Saturation %	94.1 ± 5.8	92.3 ± 3.9	94.4 ± 6.0 *
**Clinical chemistry**			
Hemoglobin (mM)	8.2 ± 1.1	8.0 ± 1.1	8.2 ± 1.1
Leukocytes (Gpt/L)	5.9 ± 0.2	7.1 ± 3.6	5.6 ± 2.8 **
Lymphocytes (Gpt/L)	1.2 ± 0.1	1.4 ± 1.4	1.2 ± 0.9
Thrombocytes (Gpt/L)	210.4 ± 74.9	213.9 ± 88.6	209.8 ± 22.1
CRP (mg/L)	35.8 ± 47.2	56.9 ± 57.8	31.5 ± 53.6 **
proBNP (mg/mL)	2052 ± 4032	2057 ± 4386	2048 ± 3880
Troponin (ng/mL)	0.034 ± 0.01	0.045 ± 0.09	0.031 ± 0.09 *
Blood glucose (mM)	8.2 ± 0.9	10.2 ± 21.1	7.5 ± 4.0
Creatinin(μM)	88.9 ± 4.0	90.5 ± 63.3	89.8 ± 74.3
GFR (mL/min/1.73m^2)	74.7 ± 1.6	74.7 ± 35.0	74.7 ± 27.0
RT-PCR (Ct)	24.9 ± 5.2	27.0 ± 5.1	24.3 ± 5.1 **
Hospitalization (d)	6.5 ± 9.8	16.1 ± 12.0	4.6 ± 8.1 **
Death (n/y)<br>% yes	325/19<br>5.5 %	51/6<br>10.5 %	274/13<br>4.5 %

Parameter		Male	
	All	untreated	treated
Number	346	39	307
Age	62.9 ± 16.5	66.9 ± 21.7	62.4 ± 15.7
**Symptoms**			

Coughing (n/y)<br>% yes	139/192<br>58.0 %	23/14<br>37.8 %	116/178 *<br>60.5 %
Dyspnea (n/y)<br>% yes	254/74<br>22.6 %	22/15<br>40.5 %	232/59 *<br>20.3 %
Fatigue (n/y)	241/86<br>26.3 %	32/5<br>13.5 %	209/81 +<br>27.9 %
Pain (n/y)<br>% yes	217/112<br>34.0 %	32/5<br>13.5 %	185/107 **<br>36.6 %
Inappetence (n/y)<br>% yes	289/39<br>11.9 %	32/5<br>13.5 %	257/34<br>11.7 %
Loss of taste and smell (n/y)<br>% yes	299/29<br>8.8 %	34/3<br>8.1 %	265/26<br>8.9 %
Diarrhoea / vomitting (n/y)<br>% yes	299/30<br>9.1 %	35/2<br>5.4 %	264/28<br>9.6 %
Fever (n/y)<br>% yes	223/105<br>32.0 %	23/14<br>37.8 %	200/91<br>31.3 %
Temperature	37.3 ± 1.0	37.4 ± 1.0	37.3 ± 1.0
Neurological Symptoms (n/y) % yes	312/16<br>4.5 %	35/2<br>5.4 %	277/14<br>4.8 %
Syncope (n/y)<br>% yes	314/14<br>4.3 %	34/3<br>8.1 %	280/11<br>3.8 %
**Preexisting illness**			
Hypertension (n/y)<br>% yes	142/189<br>57.1 %	17/21<br>55.3 %	125/168<br>57.3 %
Blood pressure systolic	129.2 ± 17.3	127.0 ± 18.1	129.4 ± 17.3
Blood pressure diastolic	79.2 ± 12.2	75.1 ± 10.3	79.5 ± 10.3
Cardiac frequency (1/min)	81.1 ± 17.0	85.3 ± 17.9	80.2 ± 16.7
Diabetes (n/y)<br>% yes	249/83<br>25.0 %	25/13<br>34.2 %	224/70<br>23.8 %
Renal insuffiency (n/y)<br>% yes	278/52<br>15.8 %	28/10<br>26.3 %	250/42 +<br>14.4 %
COPD/Asthma (n/y)<br>% yes	271/59<br>17.9 %	28/10<br>26.3 %	243/49<br>16.8 %
Active malignoma (n/y)<br>% yes	296/36 +<br>10.8 %	33/5<br>13.2 %	263/31<br>10.5 %
Inactive malignoma (n/y)<br>% yes	313/19<br>5.7 %	36/1<br>2.7 %	277/18<br>6.1 %
Immunosuppression (n/y)<br>% yes	315/16<br>4.8 %	36/2<br>5.3 %	279/14<br>4.8 %
Obesity (n/y)<br>% yes	270/58<br>17.7 %	33/4<br>10.8 %	237/54<br>18.6 %
Heart disease (n/y)<br>% yes	237/93 +<br>28.2 %	23/15<br>39.5 %	214/78<br>26.7 %
Hypothyreosis (n/y)<br>% yes	307/23 **<br>7.0 %	34/3<br>8.1 %	273/20<br>6.8 %
**Blood gas analysis**			
pO_2	10.2 ± 3.1	10.5 ± 6.4	10.1 ± 2.4
pCO_2	4.8 ± 0.7	4.7 ± 0.8	4.8 ± 0.7
O_2-Saturation %	94.0 ± 4.6	92.8 ± 6.2	94.1 ± 4.4
**Clinical chemistry**			
Hemoglobin (mM)	8.7 ± 1.4	8.5 ± 1.4	8.7 ± 1.4

Leukocytes (Gpt/L)	6.6 ± 5.6	8.0 ± 5.2	6.5 ± 5.7
Lymphocytes (Gpt/L)	1.9 ± 3.0	1.8 ± 3.2	2.0 ± 3.0
Thrombocytes (Gpt/L)	200.1 ± 81.3	191.7 ± 88.3	201.2 ± 80.4
CRP (mg/L)	43.7 ± 58.2	88.7 ± 81.4	37.5 ± 51.5 **
proBNP (mg/mL)	2278 ± 4852	2911 ± 6209	1995 ± 4142
Troponin (ng/mL)	0.060 ± 0.22	0.137 ± 0.47	0.041 ± 0.08
Blood glucose (mM)	8.0 ± 6.1	8.8 ± 4.2	7.7 ± 6.5
Creatinin(μM)	125.1 ± 132.7	142.2 ± 154.1	122.7 ± 129.5
GFR (mL/min/1.73m^2)	73.7 ± 27.7	68.8 ± 32.8	74.3 ± 26.0
RT-PCR (Ct)	24.5 ± 5.3	26.6 ± 4.6	24.2 ± 5.3 *
Hospitalization (d)	5.3 ± 8.1	11.9 ± 9.5	4.4 ± 7.4 **
Death (n/y)<br>% yes	297/21<br>6.6 %	26/11<br>29.7 %	271/10 **<br>3.6 %
