# Supplementary material for: Monoclonal Antibody Therapy for COVID-19: A Retrospective Observational Study at a Regional Hospital
Source: Infect Dis Rep. 2023 Feb 20;15(1):125–31. doi: 10.3390/idr15010013 (PMC9956015; doi:10.3390/idr15010013)
Supplement: Supplementary file 1 [file idr-15-00013-s001.zip › Table S2 CI only.pdf]

Table S2: Statistical analysis of clinical parameters for all patients treated with Casirivimab/Imdevimab. Missing data were excluded, causing different numbers for each parameter. Data for all cases can be found in Table S1. Average ± standard deviation is shown. For metric parameters significance was determined using Student´s T-test, Cross tables were analysed using Fisher´s exact test.p < 0.1: +; p < 0.05: *; p < 0.01: **. Significance signs in "All" in the female table indicate significant difference to the male sex.

**Parameter**	**All patients**		
	All	untreated	treated
Number	693	94	602
Age	64.3 ± 17.0	65.6 ± 21.2	64.1 ± 16.2
Sex (f/m)	385/308	59/37	326/271
Female %	55.6 %	61.5 %	54.6 %
**Symptoms**			
Coughing (n/y)	276/382	54/36	222/346 **
% yes	58.1 %	40.0 %	60.9 %
Dyspnea (n/y)	498/158	47/43	451/115 **
% yes	24.1 %	47.8 %	20.3 %
Fatigue (n/y)	488/166	69/21	419/145
	25.4 %	23.3 %	25.4 %
Pain (n/y)	417/238	70/20	347/218 **
% yes	36.3 %	22.2 %	38.6 %
Inappetence (n/y)	569/87	76/14	493/73
% yes	13.3 %	15.5 %	12.9 %
Loss of taste and smell (n/y)	598/57	86/4	512/53
% yes	8.7 %	4.4 %	9.4 %
Diarrhoea / vomitting (n/y)	575/81	81/9	494/72
% yes	12.3 %	10.0 %	12.7 %
Fever (n/y)	447/209	54/36	393/173 +
% yes	31.9 %	40 %	30.6 %
Temperature	37.3 ± 1.0	37.3 ± 1.0	37.3 ± 1.0
Neurological Symptoms	620/36	84/6	536/30
(n/y) % yes	5.5 %	6.7 %	5.3 %
Syncope (n/y)	629/27	80/10	549/17 **
% yes	4.1 %	11.1 %	3.0 %
**Preexisting illness**			
Hypertension (n/y)	299/357	44/49	255/308
% yes	54.4 %	52.7 %	54.7 %
Blood pressure systolic	127.9 ± 19.0	125.0 ± 22.2	128.3 ± 18.6
Blood pressure diastolic	77.9 ± 12.3	73.6 ± 11.7	78.4 ± 12.3 **
Cardiac frequency (1/min)	82.1 ± 16.1	84.3 ± 15.7	81.4 ± 16.9
Diabetes (n/y)	519/138	69/24	450/114
% yes	21.0 %	25.8 %	20.2 %
Renal insuffficency (n/y)	553/102	73/20	480/82 +
% yes	15.6 %	21.5 %	14.6 %
COPD/Asthma (n/y)	538/118	72/21	466/97
% yes	18.0 %	22.6 %	17.2 %
Active malignoma (n/y)	600/57	86/7	514/50
% yes	8.7 %	7.5 %	8.9 %
Inactive malignoma (n/y)	624/31	86/6	538/25
% yes	4.7 %	6.5 %	4.4 %

Immunosuppression (n/y)	621/35	90/3	531/32
% yes	5.3 %	3.2 %	5.7 %
Obesity (n/y)	537/117	73/19	464/98
% yes	17.9 %	20.7 %	17.4 %
Heart disease (n/y)	494/160	62/31	432/129 *
% yes	24.5 %	33.3 %	23.0 %
Hypothyreosis (n/y)	579/75	76/16	503/59 +
% yes	11.5 %	17.4 %	10.5 %
**Blood gas analysis**			
pO_2	10.2 ± 2.8	9.7 ± 4.4	10.3 ± 2.3
pCO_2	4.8 ± 0.7	4.7 ± 0.9	4.8 ± 0.7
O_2-Saturation %	94.0 ± 5.4	92.5 ± 4.9	94.3 ± 5.4 **
**Clinical chemistry**			
Hemoglobin (mM)	8.4 ± 1.3	8.2 ± 1.2	8.5 ± 1.3 +
Leukocytes (Gpt/L)	6.3 ± 4.6	7.5 ± 4.3	6.1 ± 4.6 **
Lymphocytes (Gpt/L)	1.5 ± 2.2	1.5 ± 2.2	1.6 ± 2.2
Thrombocytes (Gpt/L)	205.7 ± 77.8	204.9 ± 88.6	205,8 ± 75.8
CRP (mg/L)	39.7 ± 53.5	69.2 ± 69.3	34.3 ± 48.2 **
proBNP (mg/mL)	2297 ± 4598	2413 ± 5191	2230 ± 4243
Troponin (ng/mL)	0.05 ± 0.17	0.08 ± 0.30	0.04 ± 0.09
Blood glucose (mM)	8.2 ± 9.9	9.6 ± 16.5	7.7 ± 5.7
Creatinin(µM)	105.6 ± 109.3	111.3 ± 111.4	105.7 ± 109.0
GFR (mL/min/1.73m^2)	74.9 ± 28.1	72.4 ± 34.0	75.3 ± 27.0
PCR (Ct)	24.7 ± 5.3	26.7 ± 4.9	24.2 ± 5.3 **
Hospitalization (d)	5.7 ± 9.0	14.4 ± 11.1	4.0 ± 7.4 **
Death (n/y)	579/39	77/17	502/22 **
% yes	6.3 %	18.1 %	4.2 %

**Parameter**		**Female**	
	All	untreated	treated
Number	385	59	326
Age	66.0 ± 17.2	64.7 ± 21.0	66.2 ± 16.4
**Symptoms**			
Coughing (n/y)	145/205	31/22	114/138 **
% yes	58.6 %	41.5 %	61.6 %
Dyspnea (n/y)	262/88	25/28	237/60 **
% yes	25.1 %	52.8 %	20.2 %
Fatigue (n/y)	90/349	37/16	222/74
	25.8 %	30.2 %	25.0 %
Pain (n/y)	219/130	38/15	181/115
% yes	37.2 %	38.9 %	28.3 %
Inappetence (n/y)	297/53	44/9	253/44
% yes	15.1 %	17.0 %	14.8 %
Loss of taste and smell (n/y)	317/32	52/1	265/31 +
% yes	9.2 %	1.9 %	10.5 %
Diarrhoea / vomitting (n/y)	296/54 *	46/7	250/47
% yes	15.4 %	13.2 %	15.8 %
Fever (n/y)	240/110	31/22	209/88
% yes	31.4 %	41.5 %	29.6 %
Temperature	37.3 ± 1.0	37.2 ± 1.0	37.4 ± 1.0
Neurological Symptoms	326/24	49/4	277/20

(n/y) % yes	6.9%	7.5 %	6.7%
Syncope (n/y)	336/14	46/7	290/7 **
% yes	4.0 %	13.2 %	2.4 %
**Preexisting illness**			
Hypertension (n/y)	163/185	27/28	136/157
% yes	53.2 %	50.9 %	53.6 %
Blood pressure systolic	127.0 ± 20.5	123.9 ± 24.4	127.4 ± 20.0
Blood pressure diastolic	76.6 ± 12.2	72.8 ± 12.4	77.1 ± 12.2 *
Cardiac frequency (1/min)	82.1 ± 16.1	83.7 ± 14.5	81.6 ± 16.7
Diabetes (n/y)	286/62 *	44/11	242/51
% yes	17.8 %	20.0 %	17.4 %
Renal insufficency (n/y)	291/57	45/10	246/47
% yes	16.4 %	18.2 %	16.0 %
COPD/Asthma (n/y)	284/64	44/11	240/53
% yes	18.4 %	20.0 %	18.1 %
Active malignoma (n/y)	325/23 +	53/2	272/21
% yes	6.6 %	3.6 %	7.2 %
Inactive malignoma (n/y)	332/15	50/5	282/10 +
% yes	4.3 %	9.1 %	3.4 %
Immunosuppression (n/y)	327/21	54/1	273/20
% yes	6.0 %	1.8 %	6.8 %
Obesity (n/y)	280/68	40/15	240/53
% yes	19.5 %	27.3 %	18.1 %
Heart disease (n/y)	271/75 +	38/16	232/59
% yes	21.7 %	29.1 %	20.3 %
Hypothyreosis (n/y)	250/57 **	42/13	248/44
% yes	16.4 %	23.6 %	15.1 %
**Blood gas analysis**			
pO_2	10.2 ± 2.3	9.1 ± 2.2	10.4 ± 2.3 **
pCO_2	4.8 ± 0.7	4.8 ± 1.0	4.8 ± 0.7
O_2-Saturation (%)	94.1 ± 5.9	92.3 ± 3.9	94.5 ± 6.1 *
**Clinical chemistry**			
Hemoglobin (mM)	8.2 ± 1.1 **	8.0 ± 1.1	8.2 ± 1.1
Leukocytes (Gpt/L)	5.9 ± 3.0 *	7.1 ± 3.6	5.7 ± 2.9 **
Lymphocytes (Gpt/L)	1.3 ± 1.0 *	1.4 ± 1.3	1.2 ± 1.0
Thrombocytes (Gpt/L)	211.5 ± 75.4 *	213.2 ± 88.6	211.2 ± 72.5
CRP (mg/L)	35.8 ± 48.0 +	56.9 ± 57.8	31.2 ± 44.3 **
proBNP (mg/mL)	2255 ± 4232	2057 ± 4386	2370 ± 4172
Troponin (ng/mL)	0.04 ± 0.10	0.04 ± 0.09	0.03 ± 0.10
Blood glucose (mM)	8.3 ± 12.0	10.2 ± 21.1	7.5 ± 4.1
Creatinin(μM)	89.3 ± 73.6 **	90.5 ± 63.3	89.1 ± 75.7
GFR (mL/min/1.73m²)	75.5 ± 28.4	74.8 ± 34.9	75.7 ± 26.8
PCR (Ct)	24.9 ± 5.3	27.0 ± 5.1	24.3 ± 5.2 **
Hospitalization (d)	6.4 ± 9.8 +	16.2 ± 11.9	4.2 ± 7.8 **
Death (n/y)	304/19	51/6	253/13
% yes	5.9 %	10.5 %	4.9 %

**Parameter**		**Male**	
	All	untreated	treated
Number	308	37	271
Age	62.3 ± 14.5	66.9 ± 21.7	61.7 ± 15.6
**Symptoms**			

Coughing (n/y)<br>% yes	131/177<br>57.5 %	23/14<br>37.8 %	108/163 *<br>60.1 %
Dyspnea (n/y)<br>% yes	236/70<br>22.9 %	22/15<br>40.5 %	214/55 *<br>20.4 %
Fatigue (n/y)	229/76<br>24.9 %	32/5<br>13.5 %	197/71<br>26.5 %
Pain (n/y)<br>% yes	198/108<br>35.3 %	32/5<br>13.5 %	166/103 **<br>38.3 %
Inappetence (n/y)<br>% yes	272/34<br>11.1 %	32/5<br>13.5 %	240/29<br>10.8 %
Loss of taste and smell (n/y)<br>% yes	281/25<br>8.2 %	34/3<br>8.1 %	247/22<br>8.2 %
Diarrhoea / vomitting (n/y)<br>% yes	279/27<br>8.8 %	35/2<br>5.4 %	244/25<br>9.3 %
Fever (n/y)<br>% yes	207/99<br>32.4 %	23/14<br>37.8 %	184/85<br>31.6 %
Temperature	37.3 ± 1.0	37.4 ± 1.0	37.3 ± 1.0
Neurological Symptoms<br>(n/y) % yes	294/12<br>3.9 %	35/2<br>5.4 %	295/10<br>3.7 %
Syncope (n/y)<br>% yes	293/13<br>4.2 %	34/3<br>8.1 %	259/10<br>3.7 %
**Preexisting illness**			
Hypertension (n/y)<br>% yes	136/172<br>55.8 %	17/21<br>55.3 %	119/151<br>55.9
Blood pressure systolic	129.1 ± 17.0	127.0 ± 18.1	129.3 ± 17.0
Blood pressure diastolic	79.4 ± 12.2	75.1 ± 10.3	79.8 ± 12.3
Cardiac frequency (1/min)	82.0 ± 17.3	85.3 ± 17.9	81.2 ± 17.2
Diabetes (n/y)<br>% yes	233/76<br>24.6 %	25/13<br>34.2 %	208/63<br>23.2 %
Renal insufficency (n/y)<br>% yes	262/45<br>14.7 %	28/10<br>26.3 %	234/35 *<br>13.0 %
COPD/Asthma (n/y)<br>% yes	254/54<br>17.5 %	28/10<br>26.3 %	226/44<br>16.3 %
Active malignoma (n/y)<br>% yes	275/34<br>11.0 %	33/5<br>13.2 %	242/29<br>10.7 %
Inactive malignoma (n/y)<br>% yes	292/16<br>5.2 %	36/1<br>2.7 %	256/15<br>5.5 %
Immunosuppression (n/y)<br>% yes	294/14<br>4.5 %	36/2<br>5.3 %	258/12<br>4.4 %
Obesity (n/y)<br>% yes	257/49<br>16.0 %	33/4<br>10.8 %	224/45<br>16.7 %
Heart disease (n/y)<br>% yes	223/85<br>27.6 %	23/15<br>39.5 %	200/70<br>25.9 %
Hypothyreosis (n/y)<br>% yes	289/18<br>5.9 %	34/3<br>8.1 %	255/15<br>5.6 %
**Blood gas analysis**			
pO_2	10.2 ± 3.2	10.5 ± 6.4	10.1 ± 2.4
pCO_2	4.8 ± 0.7	4.7 ± 0.8	4.8 ± 0.6
O_2-Saturation (%)	93.9 ± 4.7	92.8 ± 6.2	94.1 ± 4.5
**Clinical chemistry**			
Hemoglobin (mM)	8.7 ± 1.4	8.5 ± 1.4	8.7 ± 1.4

Leukocytes (Gpt/L)	6.7 ± 5.8	8.0 ± 5.2	6.5 ± 5.8
Lymphocytes (Gpt/L)	1.9 ± 2.9	1.8 ± 3.2	1.9 ± 2.9
Thrombocytes (Gpt/L)	199 ± 80	191.7 ± 88.3	200.0 ± 78.9
CRP (mg/L)	44.2 ± 59.0	88.7 ± 81.4	37.6 ± 52.0 **
proBNP (mg/mL)	2355 ± 5091	2911 ± 6209	2038 ± 4380
Troponin (ng/mL)	0.06 ± 0.23	0.14 ± 0.47	0.04 ± 0.08
Blood glucose (mM)	8.1 ± 6.5	8.8 ± 4.2	7.9 ± 7.1
Creatinin(μM)	126.4 ± 136.9	142.2 ± 154.1	124.0 ± 134.3
GFR (mL/min/1.73m^2)	74.1 ± 27.9	68.8 ± 32.8	74.9 ± 27.1
Hospitalization (d)	4.9 ± 7.9	11.9 ± 9.5	3.8 ± 7.0 **
PCR (Ct)	24.5 ± 5.3	26.2 ± 4.6	24.1 ± 5.4 *
Death (n/y)<br>% yes	275/20<br>6.8 %	26/11<br>29.7 %	249/9 **<br>3.5 %
