# Supplementary material for: Monoclonal Antibody Therapy for COVID-19: A Retrospective Observational Study at a Regional Hospital
Source: Infect Dis Rep. 2023 Feb 20;15(1):125–31. doi: 10.3390/idr15010013 (PMC9956015; doi:10.3390/idr15010013)
Supplement: Supplementary file 1 [file idr-15-00013-s001.zip › Table S3 hospitalized.pdf]

Table S3: Statistical analysis of clinical parameters for hospitalized patients. Missing data were excluded, causing different numbers for each parameter. Average ± standard deviation is shown. For metric parameters, signifcance was determined using Student´s T-test, Cross tables were analysed using Fisher´s exact test. p < 0.1: +; p < 0.05: *; p < 0.01: **

Parameter	All	All patients<br>untreated	treated
Number	350	90	260
Age	67.7 ± 16.7	66.9 ± 20.9	68.0 ± 15.1
**Symptoms**			
Coughing (n/y)<br>% yes	157/179<br>53.3 %	52/31<br>37.3 %	105/148 **<br>58.5 %
Dyspnea (n/y)<br>% yes	229/107<br>31.8 %	44/39<br>47.0 %	185/68 **<br>26.9%
Fatigue (n/y)	218/117<br>34.9 %	63/20<br>24.1 %	155/97 *<br>38.5 %
Pain (n/y)<br>% yes	241/94<br>28.1 %	67/16<br>19.3 %	174/78 *<br>31.0 %
Inappetenz (n/y)<br>% yes	273/63<br>18.8 %	71/12<br>14.5 %	202/51<br>20.2 %
Loss of taste and smell (n/y)<br>% yes	309/26<br>7.8 %	79/4<br>4.8 %	230/22<br>8.7 %
Diarrhoea / vomitting (n/y)<br>% yes	291/45<br>13.4 %	74/9<br>10.8 %	217/36<br>14.2 %
Fever (n/y)<br>% yes	211/125<br>37.2 %	49/43<br>41.0 %	162/91<br>36.0 %
Temperature	37.3 ± 1.0	37.3 ± 0.9	37.3 ± 1.0
Neurological Symptoms (n/y) % yes	310/26<br>7.7 %	78/5<br>6.0 %	232/21<br>8.3 %
Syncope (n/y)<br>% yes	311/25<br>7.4 %	73/10<br>12.0 %	238/15 +<br>5.9 %
**Risk factors**			
Hypertension (n/y)<br>% yes	140/202<br>59.1 %	39/47<br>54.7 %	101/57<br>60.5 %
Blood pressure systolic	128.3 ± 20.3	124.9 ± 20.4	129.1 ± 19.6
Blood pressure diastolic	76.8 ± 12.9	73.4 ± 11.7	77.7 ± 13.1 *
Cardiac frequency (1/min)	81.8 ± 16.2	83.5 ± 15.7	81.2 ± 16.3
Diabetes (n/y)<br>% yes	254/88<br>25.7 %	62/24<br>27.9 %	192/64<br>25.0 %
Renal insufficency (n/y)<br>% yes	274/67<br>19.6 %	67/19<br>22.1 %	207/48<br>18.8 %
COPD/Asthma (n/y)<br>% yes	267/74<br>21.7 %	65/21<br>24.4 %	202/53<br>20.8 %
Active malignoma (n/y)<br>% yes	311/31<br>9.1 %	79/7<br>8.1 %	232/24<br>9.4 %
Inactive malignoma (n/y)<br>% yes	318/23<br>6.7 %	83/3<br>3.5 %	235/20<br>7.8 %
Immunosuppression (n/y)<br>% yes	262/76<br>22.5 %	68/17<br>20 %	194/59<br>23.3 %
Obesity (n/y)<br>% yes	229/109<br>32.2 %	56/30<br>34.9 %	173/79<br>31.3 %

Heart disease (n/y)<br>% yes	323/16<br>4.7 %	80/5<br>5.9 %	243/11<br>4.3 %
Hypothyreosis (n/y)<br>% yes	283/56<br>16.5 %	69/16<br>18.8 %	214/40<br>15.7 %
**Blood gas analysis**			
pO_2	10.0 ± 3.2	9.7 ± 4.5	10.1 ± 2.6
pCO_2	4.7 ± 0.8	4.7 ± 0.8	4.7 ± 0.8
O_2-Saturation %	93.5 ± 4.7	92.3 ± 4.9	94.0 ± 4.6 **
**Clinical chemistry**			
Hemoglobin (mM)	8.2 ± 1.3	8.2 ± 1.2	8.2 ± 1.3
Leukocytes (Gpt/L)	6.7 ± 5.8	7.6 ± 4.4	6.4 ± 6.2
Lymphocytes (Gpt/L)	1.3 ± 1.7	1.3 ± 1.9	1.3 ± 1.6
Thrombocytes (Gpt/L)	200.9 ± 80.9	208.4 ± 90.3	198.3 ± 77.3
CRP (mg/L)	50.4 ± 56.4	66.7 ± 69.4	43.3 ± 49.0 **
proBNP (mg/mL)	2392 ± 4728	2543 ± 5347	2308 ± 4374
Troponin (ng/mL)	0.05 ± 0.19	0.08 ± 0.3	0.04 ± 0.1
Blood glucose (mM)	8.2 ± 10.4	9.8 ± 17.0	7.5 ± 3.9
Creatinin(µM)	120.3 ± 132.8	115.1 ± 115.0	122.2 ± 139.0
GFR ($mL/min/1.73m^2$)	68.8 ± 29.1	70.1 ± 33.8	68.3 ± 27.3
RT-PCR (Ct)	24.7 ± 5.2	26.9 ± 5.1	24.2 ± 5.1 **
Hospitalization (d)	10.4 ± 9.8	14.4 ± 11.1	9.1 ± 9.0 **
Death (n/y)<br>% yes	310/34<br>9.9 %	71/16<br>18.4 %	293/18 **<br>7.0 %

Parameter	Female		
Number	All	untreated	treated
Age	185	52	133
**Symptoms**			
Coughing (n/y)<br>% yes	68.5 ± 16.8	66.8 ± 20.3	69.2 ± 15.3
Dyspnea (n/y)<br>% yes	86/90<br>51.1 %	29/18<br>38.3 %	57/72 *<br>55.8 %
Fatigue (n/y)	113/63<br>35.8 %	22/25<br>53.2 %	91/38 **<br>29.5 %
Pain (n/y)<br>% yes	111/64<br>36.6 %	32/15<br>31.9 %	79/49<br>38.3 %
Inappetenz (n/y)<br>% yes	122/53<br>30.3 %	35/12<br>25.5 %	87/41<br>32.0 %
Loss of taste and smell (n/y)<br>% yes	138/38<br>21.6 %	40/7<br>14.9 %	98/31<br>24.0 %
Diarrhoea / vomitting (n/y)<br>% yes	163/12<br>6.9 %	46/1<br>2.1 %	117/11<br>8.6 %
Fever (n/y)<br>% yes	147/29<br>16.5 %	40/7<br>14.9 %	107/22<br>17.1 %
Temperature	110/66<br>37.5 %	27/20<br>42.6 %	83/46<br>35.7 %
Neurological Symptoms (n/y) % yes	37.4 ± 0.9	37.2 ± 0.9	37.4 ± 0.9
Syncope (n/y)<br>% yes	162/14<br>8.0 %	44/3<br>6.4 %	118/11<br>8.5 %
**Preexisting illness**			

Hypertension (n/y)	161/15	40/7	121/8
% yes	8.5 %	14.9 %	6.2 %
Blood pressure systolic	79/100	22/27	57/73
	55.9 %	55.1 %	56.2 %
Blood pressure diastolic	128.5 ± 22.2	123.7 ± 24.7	130.0 ± 21.2
Cardiac frequency (1/min)	76.7 ± 13.2	72.4 ± 12.4	87.1 ± 13.2 *
Diabetes (n/y)	82.2 ± 15.4	83.1 ± 14.8	81.9 ± 15.7
% yes			
Renal insufficency (n/y)	138/41	38/11	100/30
% yes	22.9 %	22.4 %	23.1 %
COPD/Asthma (n/y)	146/33	39/10	107/23
% yes	18.4 %	20.4 %	17.7 %
Active malignoma (n/y)	145/34	38/11	107/23
% yes	19.0 %	22.4 %	17.7 %
Inactive malignoma (n/y)	168/11	47/2	121/9
% yes	6.1 %	4.1 %	6.9 %
Immunosuppression (n/y)	164/15	48/1	116/14 +
% yes	8.4 %	2.0 %	10.8 %
Obesity (n/y)	132/47	36/13	96/34
% yes	26.3 %	26.5 %	26.2 %
Heart disease (n/y)	129/49	33/16	96/33
% yes	27.5 %	32.7 %	25.6 %
Hypothyreosis (n/y)	169/8	45/4	124/4
% yes	4.5 %	8.2 %	3.1 %
**Blood gas analysis**			
pO_2	134/43	36/13	98/30
	24.3 %	26.5 %	23.4 %
pCO_2	9.7 ± 2.3	9.1 ± 2.2	10.0 ± 2.3 *
O_2-Saturation %	4.7 ± 0.8	4.7 ± 0.9	4.7 ± 0.8
**Clinical chemistry**			
Hemoglobin (mM)	8.1 ± 1.2	8.0 ± 1.1	8.1 ± 1.3
Leukocytes (Gpt/L)	6.3 ± 3.3	7.3 ± 3.7	5.8 ± 3.1 *
Lymphocytes (Gpt/L)	1.1 ± 0.5	1.2 ± 0.5	1.1 ± 0.5
Thrombocytes (Gpt/L)	203.8 ± 71.7	219.2 ± 91.2	194.4 ± 91.6
CRP (mg/L)	44.0 ± 47.7	58.9 ± 59.6	37.6 ± 40.3 *
proBNP (mg/mL)	2173 ± 4271	2234 ± 4550	2139 ± 4156
Troponin (ng/mL)	0.04 ± 0.10	0.05 ± 0.09	0.03 ± 0.11
Blood glucose (mM)	8.7 ± 13.7	10.5 ± 22.1	7.7 ± 4.5
Creatinin(µM)	91.6 ± 62.7	94.3 ± 65.9	90.5 ± 61.6
GFR (mL/min/1.73m²)	70.8 ± 28.7	71.0 ± 34.5	70.7 ± 26.1
RT-PCR (Ct)	24.7 ± 5.1	27.2 ± 5.3	24.0 ± 4.8 **
Hospitalization (d)	11.1 ± 10.6	16.2 ± 11.9	9.2 ± 9.4 **
Death (n/y)	165/16	45/6	120/10
% yes	11.0 %	11.8 %	7.7 %

Parameter		Male	
	All	untreated	treated
Number	165	38	127
Age	66.8 ± 16.7	66.9 ± 22.0	66.8 ± 15.0
**Symptoms**			
Coughing (n/y)	71/89	23/13	48/76 *

% yes	55.6 %	36.1 %	61.3 %
Dyspnea (n/y)<br>% yes	116/44<br>27.5 %	22/14<br>38.9 %	94/30 +<br>24.2 %
Fatigue (n/y)	107/53<br>33.1 %	31/5<br>13.9 %	76/48 **<br>38.7 %
Pain (n/y)<br>% yes	119/41<br>25.6 %	32/4<br>11.1 %	37/37 *<br>29.8 %
Inappetenz (n/y)<br>% yes	135/25<br>15.6 %	31/5<br>13.9 %	104/20<br>16.1 %
Loss of taste and smell (n/y)<br>% yes	146/14<br>8.8 %	33/3<br>8.3 %	113/11<br>8.9 %
Diarrhoea / vomitting (n/y)<br>% yes	144/16<br>10.0 %	34/2<br>5.6 %	110/14<br>11.3 %
Fever (n/y)<br>% yes	101/59<br>36.9 %	22/14<br>38.9 %	79/45<br>36.3 %
Temperature	37.2 ± 1.0	37.4 ± 1.0	37.2 ± 1.0
Neurological Symptoms (n/y) % yes	148/12<br>7.5 %	34/2<br>5.6 %	114/10<br>8.1 %
Syncope (n/y)<br>% yes	150/10<br>6.3 %	33/3<br>8.3 %	117/7<br>5.6 %
**Risk factors**			
Hypertension (n/y)<br>% yes	61/102<br>62.6 %	17/20<br>54.1 %	44/82<br>65.1 %
Blood pressure systolic	128.1 ± 17.8	127.0 ± 18.1	128.3 ± 17.8
Blood pressure diastolic	76.9 ± 12.6	75.1 ± 10.3	77.2 ± 13.0
Cardiac frequency (1/min)	81.3 ± 17.2	84.3 ± 17.5	80.4 ± 17.1
Diabetes (n/y)<br>% yes	116/47<br>28.8 %	24/13<br>25.1 %	92/34<br>27.0 %
Renal insufficency (n/y)<br>% yes	128/34<br>21.0 %	28/9<br>24.3 %	100/25<br>20.0 %
COPD/Asthma (n/y)<br>% yes	122/40<br>24.7 %	27/10<br>27.0 %	95/30<br>24.0 %
Active malignoma (n/y)<br>% yes	143/20<br>12.3 %	32/5<br>13.5 %	111/15<br>11.9 %
Inactive malignoma (n/y)<br>% yes	154/8<br>4.9 %	35/2<br>5.4 %	119/6<br>4.8 %
Immunosuppression (n/y)<br>% yes	130/29 +<br>18.2 %	32/4<br>11.1 %	98/25<br>20.3 %
Obesity (n/y)<br>% yes	100/60<br>37.5 %	23/14<br>37.8 %	77/46<br>37.4 %
Heart disease (n/y)<br>% yes	154/8<br>4.9 %	35/1<br>2.8 %	119/7<br>5.6 %
Hypothyreosis (n/y)<br>% yes	149/13 *<br>8.0 %	33/3<br>8.3 %	116/10<br>7.9 %
**Blood gas analysis**			
pO_2	10.3 ± 3.7 +	10.5 ± 6.5	10.3 ± 2.8
pCO_2	4.7 ± 0.8	4.7 ± 0.8	4.7 ± 0.8
O_2-Saturation %	93.5 ± 5.4	92.6 ± 6.1	93.8 ± 5.1
**Clinical chemistry**			
Hemoglobin (mM)	8.4 ± 1.4 +	8.4 ± 1.4	8.4 ± 1.4

Leukocytes (Gpt/L)	7.3 ± 7.7	8.0 ± 5.3	7.0 ± 8.3
Lymphocytes (Gpt/L)	1.6 ± 2.4 *	1.6 ± 3.0	1.6 ± 2.2
Thrombocytes (Gpt/L)	197.6 ± 90.5	192.7 ± 89.4	199.3 ± 91.1
CRP (mg/L)	57.9 ± 64.4 *	85.4 ± 80.1	49.4 ± 56.4 *
proBNP (mg/mL)	2710 ± 5343	2949 ± 6340	2567 ± 4729
Troponin (ng/mL)	0.08 ± 0.26	0.14 ± 0.48	0.06 ± 0.09
Blood glucose (mM)	7.7 ± 3.5	8.8 ± 4.2	7.2 ± 3.0 *
Creatinin(μM)	153.0 ± 177.3 **	143.5 ± 156.0	156.1 ± 184.2
GFR (mL/min/1.73m^2)	66.4 ± 29.5	68.7 ± 33.2	65.6 ± 28.3
RT-PCR (Ct)	24.7 ± 5.3	26.4 ± 4.9	24.4 ± 5.4 +
Hospitalization (d)	9.6 ± 8.8	11.9 ± 9.5	8.9 ± 8.6 +
Death (n/y)<br>% yes	145/18<br>11.0 %	26/10<br>27.8 %	119/8 **<br>6.3 %
