# Supplementary material for: Monoclonal Antibody Therapy for COVID-19: A Retrospective Observational Study at a Regional Hospital
Source: Infect Dis Rep. 2023 Feb 20;15(1):125–31. doi: 10.3390/idr15010013 (PMC9956015; doi:10.3390/idr15010013)
Supplement: Supplementary file 1 [file idr-15-00013-s001.zip › Table S4 CI only hospitalized.pdf]

Table S4: Statistical analysis of clinical parameters for hospitalized patients treated with Casirivimab/Imdevimab. Missing data were excluded, causing different numbers for each parameter. Average ± standard deviation is shown. For metric parameters, significance was determined using Student´s T-test, Cross tables were analysed using Fisher´s exact test. p < 0.1: +; p < 0.05: *; p < 0.01: **. Significance signs in "All" in the female table indicate significant difference to the male sex.

Parameter	All patients		
	All	untreated	treated
Number	315	90	225
Age	67.1 ± 17.0	66.9 ± 20.9	67.2 ± 15.2
**Symptoms**			
Coughing (n/y)	146/156	52/31	94/125 **
% yes	51.7 %	37.3 %	57.1 %
Dyspnea (n/y)	207/95	44/39	163/56 **
% yes	31.5 %	44.0 %	25.6 %
Fatigue (n/y)	201/100	63/20	138/80 *
	33.2 %	24.1 %	36.7 %
Pain (n/y)	214/87	67/16	147/71 *
% yes	28.9 %	19.3 %	32.6 %
Inappetenz (n/y)	248/54	71/12	177/42
% yes	17.9 %	14.5 %	19.2%
Loss of taste and smell (n/y)	278/23	79/4	199/19
% yes	7.6 %	4.8 %	8.7 %
Diarrhoea / vomitting (n/y)	263/39	74/9	189/30
% yes	12.9 %	10.8 %	13.7 %
Fever (n/y)	189/113	49/34	140/79
% yes	37.4 %	41.0 %	36.1 %
Temperature	37.3 ± 1.0	37.3 ± 1.0	37.3 ± 1.0
Neurological Symptoms	282/20	78/5	204/15
(n/y) % yes	6.6 %	6.0 %	6.8 %
Syncope (n/y)	280/22	73/10	207/12 +
% yes	7.3 %	12.0 %	5.5 %
**Risk factors**			
Hypertension (n/y)	129/178	39/47	90/131
% yes	58.0 %	54.7 %	59.3 %
Blood pressure systolic	127.7 ± 20.1	124.9 ± 22.4	128.5 ± 19.3
Blood pressure diastolic	67.6 ± 12.8	73.4 ± 11.7	77.5 ± 13.1
Cardiac frequency (1/min)	82.3 ± 16.6	83.5 ± 15.7	81.7 ± 17.0
Diabetes (n/y)	232/75	62/24	170/51
% yes	24.4 %	27.9 %	23.1 %
Renal insuffficency (n/y)	248/58	67/19	181/39
% yes	19.0 %	22.1 %	17.7 %
COPD/Asthma (n/y)	241/65	65/21	176/44
% yes	21.2 %	24.4 %	20.0 %
Active malignoma (n/y)	279/28	79/7	200/21
% yes	9.1 %	8.1 %	9.5 %
Inactive malignoma (n/y)	292/14	80/5	212/9
% yes	4.6 %	5.9 %	4.1 %
Immunosuppression (n/y)	287/19	83/3	204/16
% yes	6.2 %	3.5 %	7.3 %

Obesity (n/y)	240/64	68/17	172/47
% yes	21.1 %	20.0 %	21.5 %
Heart disease (n/y)	208/98	56/30	152/68
% yes	32.0 %	34.9 %	30.9 %
Hypothyreosis (n/y)	261/45	69/16	192/29
% yes	14.7 %	18.8 %	13.1 %
**Blood gas analysis**			
pO_2	10.0 ± 3.3	9.7 ± 4.5	10.2 ± 2.6
pCO_2	4.7 ± 0.8	4.7 ± 0.8	4.7 ± 0.8
O_2-Saturation %	93.6 ± 4.9	92.3 ± 4.9	94.1 ± 4.8
**Clinical chemistry**			
Hemoglobin (mM)	8.3 ± 1.3	8.2 ± 1.2	8.3 ± 1.3
Leukocytes (Gpt/L)	6.9 ± 6.1	7.6 ± 4.4	6.6 ± 6.4
Lymphocytes (Gpt/L)	1.3 ± 1.5	1.3 ± 1.9	1.2 ± 1.2
Thrombocytes (Gpt/L)	200.8 ± 80.6	208.4 ± 90.3	197.6 ± 76.1
CRP (mg/L)	52.0 ± 57.9	69.7 ± 69.5	44.4 ± 50.4
proBNP (mg/mL)	2562 ± 4965	2543 ± 5348	2575 ± 4713
Troponin (ng/mL)	0.06 ± 0.20	0.08 ± 0.31	0.04 ± 0.11
Blood glucose (mM)	8.4 ± 11.1	9.8 ± 17.0	7.6 ± 4.2
Creatinin(µM)	121.9 ± 139.8	115.1 ± 115.0	124.8 ± 149.4
GFR (mL/min/1.73m^2)	69.7 ± 29.5	70.1 ± 33.8	69.5 ± 27.5
PCR (Ct)	24.8 ± 5.0	26.7 ± 4.9	24.0 ± 4.9
Hospitalization (d)	10.3 ± 9.9	14.4 ± 11.1	8.6 ± 8.9 **
Death (n/y)	276/33	71/16	205/17 *
% yes	10.7 %	18.3 %	7.7 %

**Parameter**		**Female**	
	All	untreated	treated
Number	169	52	117
Age	68.1 ± 17.0	66.8 ± 20.3	68.6 ± 15.5
**Symptoms**			
Coughing (n/y)	82/78	29/18	53/60
% yes	48.8 %	38.3 %	53.1 %
Dyspnea (n/y)	105/55	22/25	83/30 **
% yes	34.4 %	53.2 %	26.5 %
Fatigue (n/y)	103/56	32/15	71/41
	35.2 %	31.9 %	36.6 %
Pain (n/y)	111/48	35/12	76/36
% yes	30.2 %	35.5 %	32.1 %
Inappetenz (n/y)	127/33	40/7	87/26
% yes	20.6 %	14.9 %	23.0 %
Loss of taste and smell (n/y)	147/12	46/1	101/11
% yes	7.5 %	2.1 %	9.8 %
Diarrhoea / vomitting (n/y)	135/25	40/7	95/18
% yes	15.6 %	14.9 %	15.9 %
Fever (n/y)	100/60	27/20	73/40
% yes	37.5 %	42.6 %	35.4 %
Temperature	37.4 ± 1.0	37.2 ± 0.9	37.4 ± 1.0
Neurological Symptoms (n/y) % yes	148/12<br>7.5%	44/3<br>6.4 %	104/9<br>8.0 %

Syncope (n/y)	147/13	40/7	107/6 +
% yes	8.1 %	14.9 %	5.3 %
**Preexisting illness**			
Hypertension (n/y)	72/91	22/27	50/64
% yes	55.8 %	55.1 %	56.1 %
Blood pressure systolic	127.6 ± 22.4	123.7 ± 24.7	129.1 ± 21.4
Blood pressure diastolic	76.2 ± 13.0	72.4 ± 12.4	77.6 ± 13.0 *
Cardiac frequency (1/min)	82.2 ± 16.0	83.1 ± 14.8	81.8 ± 16.6
Diabetes (n/y)	129/34	38/11	91/23
% yes	20.9 %	22.4 %	22.2 %
Renal insuffiency (n/y)	134/29	39/10	95/19
% yes	17.8 %	20.4 %	16.7 %
COPD/Asthma (n/y)	133/30	38/11	95/19
% yes	18.4 %	22.4 %	16.7 %
Active malignoma (n/y)	154/9 *	47/2	107/7
% yes	5.5 %	4.1 %	6.1 %
Inactive malignoma (n/y)	155/8	45/4	110/4
% yes	4.9 %	8.2 %	3.5 %
Immunosuppression (n/y)	150/13	48/1	102/12
% yes	8.0 %	2.0 %	10.5 %
Obesity (n/y)	122/41 +	36/13	86/28
% yes	25.2 %	26.5 %	24.6 %
Heart disease (n/y)	118/45 +	33/16	85/29
% yes	27.6 %	32.7 %	25.4 %
Hypothyreosis (n/y)	126/37 **	35/13	90/24
% yes	22.7 %	26.5 %	21.1 %
**Blood gas analysis**			
pO_2	9.8 ± 2.4	9.1 ± 2.2	10.1 ± 2.4 **
pCO_2	4.7 ± 0.8	4.7 ± 0.9	4.7 ± 0.8
O_2-Saturation %	93.6 ± 4.2	92.1 ± 4.0	94.3 ± 4.1 **
**Clinical chemistry**			
Hemoglobin (mM)	8.1 ± 1.2 +	8.0 ± 1.1	8.2 ± 1.3
Leukocytes (Gpt/L)	6.4 ± 3.4	7.3 ± 3.7	6.0 ± 3.2 *
Lymphocytes (Gpt/L)	1.1 ± 0.5	1.2 ± 0.5	1.1 ± 0.5
Thrombocytes (Gpt/L)	206.3 ± 73.0	219.2 ± 90.2	200.1 ± 62.5
CRP (mg/L)	44.7 ± 49.1 *	58.9 ± 59.6	37.7 ± 41.7 *
proBNP (mg/mL)	2400 ± 4497	2239 ± 4550	2503 ± 4505
Troponin (ng/mL)	0.04 ± 0.11	0.05 ± 0.09	0.03 ± 0.12
Blood glucose (mM)	8.9 ± 14.4	10.5 ± 22.1	7.8 ± 4.3
Creatinin(µM)	90.3 ± 64.1 **	94.3 ± 65.9	88.4 ± 63.4
GFR (mL/min/1.73m^2)	72.2 ± 28.8	71.0 ± 34.5	72.3 ± 25.7
Hospitalization (d)	11.0 ± 10.7	16.2 ± 11.9	8.8 ± 9.3 **
PCR (Ct)	25.2 ± 5.1	27.0 ± 5.1	24.3 ± 4.8 **
Death (n/y)	149/16	45/6	104/10
% yes	9.7 %	11.8 %	8.8 %

Parameter		Male	
	All	untreated	treated
Number	146	38	108
Age	66.1 ± 16.9	66.9 ± 22.0	65.8 ± 14.9
**Symptoms**			

Coughing (n/y)<br>% yes	64/78<br>54.9 %	23/13<br>36.1 %	41/65 *<br>61.3 %
Dyspnea (n/y)<br>% yes	102/40<br>28.2 %	22/14<br>38.9 %	80/26<br>24.5 %
Fatigue (n/y)<br>% yes	98/44<br>31.0 %	31/5<br>13.9 %	67/39 *<br>36.8 %
Pain (n/y)<br>% yes	103/39<br>27.5 %	32/4<br>11.1 %	71/35 *<br>33.0 %
Inappetenz (n/y)<br>% yes	121/21<br>14.8 %	31/5<br>13.9 %	90/16<br>15.1 %
Loss of taste and smell (n/y)<br>% yes	131/11<br>7.7 %	33/3<br>8.3 %	98/8<br>7.5 %
Diarrhoea / vomitting (n/y)<br>% yes	128/14<br>9.9 %	34/2<br>5.6 %	94/12<br>11.3 %
Fever (n/y)<br>% yes	89/53<br>37.3 %	22/14<br>38.9 %	67/39<br>36.8 %
Temperature	37.2 ± 1.0	37.0 ± 1.0	37.2 ± 1.1
Neurological Symptoms (n/y) % yes	134/8<br>5.6 %	34/2<br>5.6 %	100/6<br>5.7 %
Syncope (n/y)<br>% yes	133/9<br>6.3 %	33/3<br>8.3 %	100/6<br>5.7 %
**Risk factors**			
Hypertension (n/y)<br>% yes	57/87<br>60.4 %	17/20<br>54.1 %	40/67<br>62.6 %
Blood pressure systolic	127.7 ± 17.0	127.0 ± 18.1	127.8 ± 16.9
Blood pressure diastolic	77.0 ± 12.7	75.1 ± 10.3	77.5 ± 13.2
Cardiac frequency (1/min)	82.4 ± 17.6	84.3 ± 17.5	81.7 ± 17.7
Diabetes (n/y)<br>% yes	103/41<br>28.5 %	24/13<br>25.1 %	79/28<br>26.2 %
Renal insufficency (n/y)<br>% yes	114/29<br>20.3 %	28/9<br>24.3 %	86/20<br>18.9 %
COPD/Asthma (n/y)<br>% yes	108/35<br>24.5 %	27/10<br>27.0 %	81/25<br>23.6 %
Active malignoma (n/y)<br>% yes	125/19<br>13.2 %	32/5<br>13.5 %	93/14<br>13.1 %
Inactive malignoma (n/y)<br>% yes	135/8<br>5.6 %	33/3<br>8.3 %	102/5<br>4.7 %
Immunosuppression (n/y)<br>% yes	137/6<br>4.2 %	35/2<br>5.4 %	102/4<br>3.8 %
Obesity (n/y)<br>% yes	118/23<br>16.3 %	32/4<br>11.1 %	86/19<br>18.1 %
Heart disease (n/y)<br>% yes	90/53<br>37.1 %	23/14<br>37.8 %	67/39<br>36.8 %
Hypothyreosis (n/y)<br>% yes	137/6<br>4.2 %	35/1<br>2.8 %	102/5<br>4.7 %
**Blood gas analysis**			
pO_2	10.4 ± 4.1	10.5 ± 6.5	10.3 ± 2.8
pCO_2	4.7 ± 0.8	4.7 ± 0.8	4.7 ± 0.8
O_2-Saturation %	93.4 ± 5.6	92.6 ± 6.1	93.8 ± 5.4
**Clinical chemistry**			
Hemoglobin (mM)	8.4 ± 1.4	8.4 ± 1.4	8.4 ± 1.4
Leukocytes (Gpt/L)	7.4 ± 8.1	8.0 ± 5.3	7.1 ± 8.9

Lymphocytes (Gpt/L)	1.4 ± 2.1	1.6 ± 3.0	1.4 ± 1.6
Thrombocytes (Gpt/L)	194.3 ± 88.6	192.7 ± 89.4	194.8 ± 88.7
CRP (mg/L)	60.7 ± 66.0	85.4 ± 80.1	51.6 ± 57.8 **
proBNP (mg/mL)	2808 ± 5638	2948 ± 6339	2695 ± 5117
Troponin (ng/mL)	0.08 ± 0.28	0.14 ± 0.48	0.06 ± 0.10
Blood glucose (mM)	7.9 ± 3.7	8.8 ± 4.2	7.3 ± 3.2 +
Creatinin(μM)	159.1 ± 187.9	153.5 ± 156.0	165.1 ± 199.4
GFR (mL/min/1.73m^2)	66.7 ± 30.2	68.7 ± 33.2	65.9 ± 29.1
PCR (Ct)	24.4 ± 5.0	26.2 ± 4.7	23.7 ± 5.0 *
Hospitalization (d)	9.4 ± 8.7	11.9 ± 9.5	8.5 ± 8.3 *
Death (n/y)<br>% yes	127/17<br>11.8 %	26/10<br>27.8 %	101/7 **<br>6.5 %
